# Classification of the nucleolytic ribozymes based upon catalytic mechanism

**DOI:** 10.12688/f1000research.19324.1

**Published:** 2019-08-19

**Authors:** David M.J. Lilley

**Affiliations:** 1 Cancer Research UK Nucleic Acid Structure Research Group, MSI/WTB Complex, The University of Dundee, Dundee, UK; 2 School of Life Sciences, Xiamen University, Xiamen, China

**Keywords:** Nucleolytic ribozymes, general acid-base catalysis, metal ions

## Abstract

The nucleolytic ribozymes carry out site-specific RNA cleavage reactions by nucleophilic attack of the 2′-oxygen atom on the adjacent phosphorus with an acceleration of a million-fold or greater. A major part of this arises from concerted general acid–base catalysis. Recent identification of new ribozymes has expanded the group to a total of nine and this provides a new opportunity to identify sub-groupings according to the nature of the general base and acid. These include nucleobases, hydrated metal ions, and 2′-hydroxyl groups. Evolution has selected a number of different combinations of these elements that lead to efficient catalysis. These differences provide a new mechanistic basis for classifying these ribozymes.

Ribozymes are enzymes that are built from RNA rather than from protein. Although they plausibly played a key role in the emergence of primitive life early in the history of the planet, some are still with us, catalyzing some extremely important cellular reactions such as the condensation of amino acids to form polypeptides in the core of the ribosome. Therefore, understanding how RNA could catalyze cellular reactions is important and recently this field has seen considerable progress that is reviewed here. This review focuses on a sub-group of the ribozymes where some general principles can be discerned.

## The nucleolytic ribozymes

The nucleolytic ribozymes are relatively small RNAs (all but one have fewer than 100 nucleotides) that bring about site-specific cleavage of the backbone ^1, 2^. In some ribozymes, the reverse (ligation) reaction can occur ^3– 8^. In the protein enzymes, such phosphoryl transfer reactions are catalyzed in one of two ways, either by the use of metal ions to organize and activate the active center or by general acid–base catalysis using histidine. RNA has a small fraction of the chemical resources of a protein. It has only four similar heterocyclic nucleobases, the 2′-hydroxyl group, and bound hydrated metal ions. Although some nucleolytic ribozymes certainly employ hydrated metal ions in their catalysis, the common theme among these species is the use of concerted general acid–base catalysis. Evolution has found a variety of ways of employing the limited resources of RNA in different combinations to accelerate the reactions by around one million-fold or more and it is this feat of chemical catalysis that we wish to understand.

There are now nine members within this group, and they have distinct folds that are generally based on helical junctions or pseudoknot structures or both. The hammerhead ^9, 10^, hairpin ^3, 11, 12^, Varkud satellite (VS) ^13^, and hepatitis delta virus (HDV) ^14– 16^ ribozymes have been known for several decades, but more recently the group has expanded significantly thanks to the work of the Breaker lab, starting with the *glmS* ribozyme ^17^. More recently, their bioinformatic pipeline ^18^ has added four more distinct species: the twister, TS, pistol, and hatchet ribozymes ^19^. This complete group ( Table 1) now provides a new opportunity to compare structures and mechanisms and to contrast and perceive sub-groupings.

**Table 1.** General bases and acids participating in the cleavage reactions of the nucleolytic ribozymes.

Group	Ribozyme	Base	Acid
G + A			
	Hairpin	G N1	A N1
	VS	G N1	A N1
G + A(N3)			
	Twister	G N1	A N3
M + C			
	HDV	M ^2+^ OH ^−^	C N3
	TS	M ^2+^ OH ^−^	C N3?
G O2′			
	Hammerhead	G N1	O2′
G + M			
	Pistol	G N1	M ^2+^ H _2_O
G + coenzyme			
	*glmS*	G N1	Glc6P amine

The groups are named by the general base followed by the general acid in the cleavage reaction. M designates a hydrated divalent metal ion directly participating in catalysis. HDV, hepatitis delta virus; VS, Varkud satellite; Glc6P, glucosamine-6-phosphate

## Mechanism and catalysis of phosphoryl transfer

**Figure 1.**
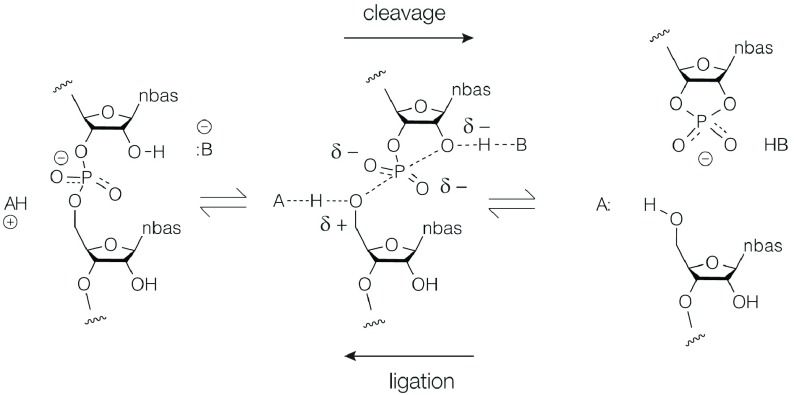
The chemical mechanism of the nucleolytic ribozymes, showing catalysis by general acid–base catalysis. The cleavage reaction is left to right and the ligation reaction right to left. The central phosphorane structure will be close to the transition state in the reaction trajectory. In the cleavage reaction, B ^−^ acts as a general base to deprotonate the O2′ nucleophile, and the general acid AH ^+^ protonates the 5′-oxyanion leaving group.

The standard reaction carried out by the nucleolytic ribozymes is shown in Figure 1. The cleavage reaction (left to right) is brought about by nucleophilic attack of the O2′ on its adjacent phosphate group. The S _N_2 reaction passes through a trigonal bipyramidal phosphorane intermediate ^20^ that is probably close to the transition state that decomposes by departure of the O5′to leave the cyclic 2′3′phosphate ^21^. The reaction is typically accelerated by at least a million-fold by the ribozymes, although the estimation requires knowledge of the baseline rate of the uncatalyzed reaction which is not easy to estimate. If the products are held in place by the RNA structure, then the reverse ligation reaction (right to left) can also occur, as it does, for example, with the hammerhead and hairpin ribozymes.

There are four potential elements to the catalysis ^22– 24^. First, if the RNA can facilitate a near in-line geometry of attack, this will provide a small contribution to rate enhancement. Second, the phosphorane could be stabilized by a combination of hydrogen bonding and electrostatics. Third, conversion of the 2′OH to an alkoxide by a general base will generate a much stronger nucleophile. Lastly, protonation of the O5′ by a general acid will create a better leaving group. The last two jointly constitute concerted general acid–base catalysis and undoubtedly make the largest contribution to the catalytic rate enhancement ^25^. Of course, ultimately, all catalytic strategy can be reduced to transition state stabilization, and general acid–base catalysis also reduces charge separation in the transition state.

The nucleolytic ribozymes use a variety of functionalities in different combinations to perform general acid–base catalysis. Nucleobases are used most frequently, and G, A, and C are all employed in different ribozymes. They suffer from the disadvantage that their natural p *K*_a_ values are not close to neutrality. The observed rate of cleavage ( *k*_obs_) will be related to the intrinsic rate ( *k*_cat_) by:

*k*_obs_ = *k*_cat_ . *f*_A_ . *f*_B_

*f*_A_ and *f*_B_ are the fractions of protonated acid and deprotonated base, respectively, at any given pH. For example, if a ribozyme uses a general base and acid of p *K*_a_ = 9 and 5, respectively (values close to those of a typical G+A ribozyme), then *f*_A_ . *f*_B_ will be 10 ^−4^ at neutrality. Application of this to the maximum rates observable for the VS ribozyme gives a *k*_cat_ that is not greatly slower than that of ribonuclease A ^26^. By shifting p *K*_a_ values closer to neutrality and thus raising *f*_A_. *f*_B_, faster rates can be obtained. In an electronegative environment of RNA, it is easy to see how the p *K*_a_ of adenine or cytosine can be raised and an extreme case of this is observed in the twister ribozyme (see the “G + A(N3): the twister ribozyme” section below). Lowering a higher p *K*_a_ value will likely require juxtaposition of a metal ion. It should be noted that although the fraction of catalyst that is in the correct state of protonation is important, there is a degree of compensation between this and the reactivity of the groups undergoing proton transfer ^27^.

## General base catalysis by guanine nucleobases

The most common element in the mechanisms of the nucleolytic ribozymes is a guanine nucleobase acting as a general base, which is true for the all the ribozymes except the group containing HDV and TS. The guanine nucleobase acts rather like the analog of the imidazole group of histidine in protein enzymes and accepts the proton from the O2′ nucleophile at its N1 position. In order to do this, it must be in its deprotonated form, and in each case this has been demonstrated by the pH dependence of the cleavage reaction, generally combined with atomic mutagenesis. The normal p *K*_a_ of guanine is around 9.5; but for maximum catalytic efficiency, this would ideally be lowered. In the VS ribozyme, this has been measured at 8.4 ^28^, where proximity of a bound metal ion likely reduces the p *K*_a_.

## Grouping the nucleolytic ribozymes

**Figure 2.**
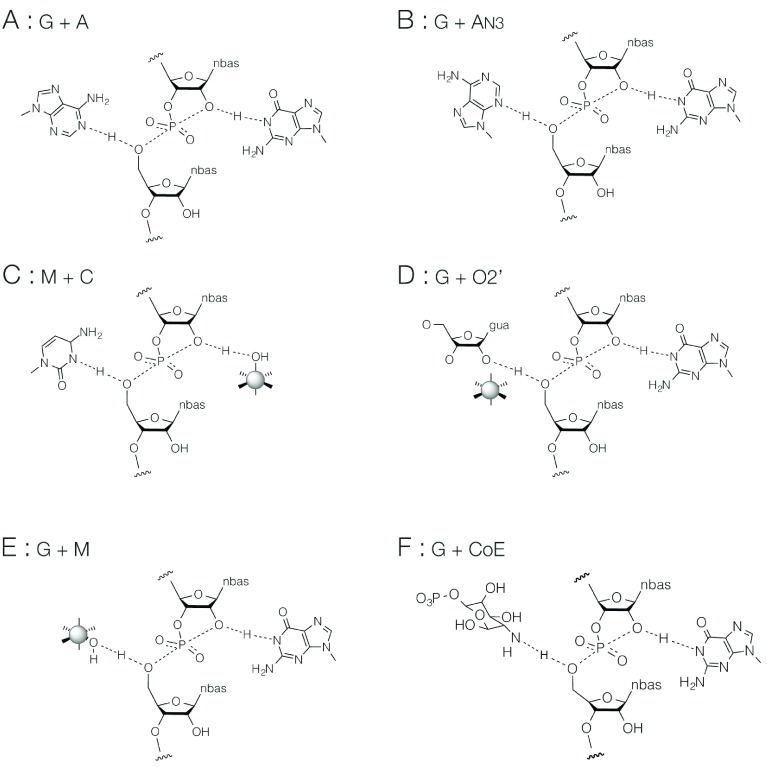
The six mechanisms observed in the nucleolytic ribozymes, classified according to the general base and acid used. For clarity, charges are not shown in these depictions. The groupings correspond to those listed in Table 1. CoE refers to the use of an exogenous coenzyme; *glmS* binds glucosamine-6-phosphate to act as a general acid.

While guanine acting as general base is very common, the general acid is rather more variable. This is a nucleobase in (probably) five cases, in three of which it is adenine. But it is also frequently not a nucleobase. Consideration of the general base and acid provides a way to group the nucleolytic ribozymes mechanistically ( Table 1 and Figure 2).

## G + A: the hairpin and VS ribozymes

These ribozymes use the N1 of a guanine and an adenine in proton transfer. The role of an adenine nucleobase as a general acid has been proven by phosphorothiolate substitution in the hairpin ^29^ and VS ^26^ ribozymes, and despite having very different overall folds, the active centers of the hairpin and VS ribozymes are closely similar. Both are generated by interaction of two internal loops, and the disposition of the guanine general base, adenine general acid, and scissile phosphate is the same for both ^26, 30, 31^. Both ribozymes exhibit activity in high concentrations of monovalent ions as the sole ion ^32^ and thus there is no evidence for a direct role for metal ions in these ribozymes, although some electrostatic stabilization of the transition state is possible. In order for adenine to act as a general acid, it must be protonated at N1. Thus, the concerted general acid–base catalysis requires an unprotonated guanine and a protonated adenine ( Figure 2A). Although the p *K*_a_ of unperturbed adenine is 4 or lower, these values are typically raised in the electronegative context of the folded RNA. For example, that measured in the VS ribozyme is 5.2 ^28^. The pH profile for the VS ribozyme is bell-shaped, reflecting the requirement for an unprotonated guanine acting as a general base and a protonated adenine acting as a general acid. By the principle of microscopic reversibility, the ligation reaction will proceed via the same chemical mechanism in reverse, so that neutral adenine will act as the general base deprotonating the O5′ nucleophile, and neutral guanine will act as the general acid protonating the O2′ leaving group.

## G + A(N3): the twister ribozyme

**Figure 3.**
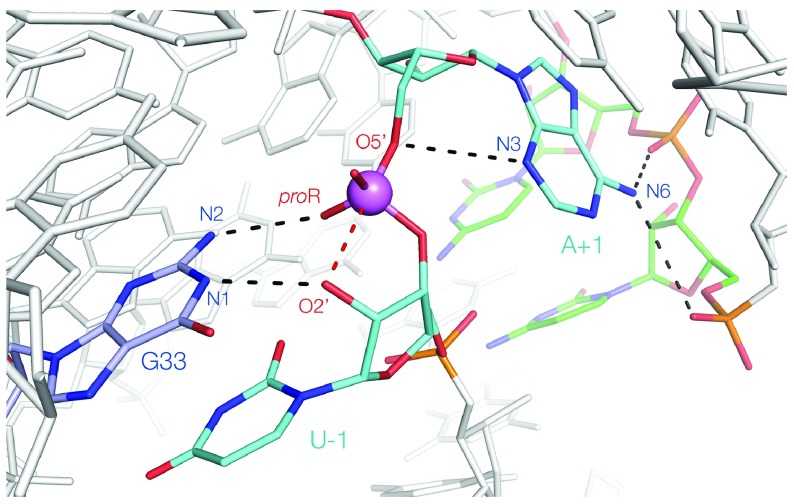
The proposed structure of the active site of the twister ribozyme. This is derived from the crystal structure, with a rotation of U-1 that brings the O2′ (modeled) into an in-line position ^34^. G33 both acts as a general base (accepting a proton at N1) and stabilizes the transition state (N2 donates a proton to the *pro*R non-bridging O atom of the scissile phosphate). A+1 acts as a general acid, donating a proton from N3. Its N6 donates protons to successive phosphates in the backbone, thereby raising its p *K*_a_.

The twister ribozyme ^33^ is a third G+A ribozyme but with two significant differences. It was the first of the group of new ribozymes, and three crystal structures have been determined, showing that the fold is a unique inverted double pseudoknot ^34– 36^. As a result of extensive mechanistic studies ^24^ in addition to the structural analysis, it is arguably the best understood of the nucleolytic ribozymes. Although none of the structures is well aligned for nucleophilic attack (and the one that was closest has other significant disruption of the structure ^36^), it was easy to see how to rotate the 5′ nucleotide in the active center to bring it into an in-line geometry ^34, 37^. Moreover, this had the effect of bringing the guanine G33 that was known to act as a general base into position in addition to stabilizing the transition state by bonding its N2 to the scissile phosphate ( Figures 2B and Figure 3). So with three catalytic strategies identified, what is the general acid? Surprisingly, it turned out to be the adenine immediately 3′ to the scissile phosphate (A+1). Moreover, it was not the usual N1 that acts in proton transfer (in fact, geometrically, this is not possible given its position) but rather the much more acidic N3. This was confirmed by deaza substitution ^24^. Microscopic p *K*_a_ values of the N1 and N3 atoms indicate that a proton will be 100-fold more likely to reside on N1 rather than N3 ^38^ yet this is partially compensated by the unusual environment in the active center. The N6 of A+1 donates both its protons to successive phosphate groups. The net effect of this is to raise the p *K*_a_ of A+1 to neutrality, thus offsetting its low probability of protonation at N3. The twister is thus another G+A ribozyme but with a difference. The active site shows how evolution has generated a perfect environment for catalysis of the transesterification, comprising in-line attack, transition state stabilization, and concerted general acid–base catalysis ( Figure 3). An alternative mechanism has been proposed for the twister ribozyme; for a neutral perspective on this, the reader is directed to an excellent critical evaluation by Breaker ^39^ and a very recent computational study ^40^.

## M ^2+^ + C: metal ions as the general base and cytosine as the general acid: the HDV and TS ribozymes

Although the hairpin and VS ribozymes have been shown to retain some activity at pH = 8 in the presence of 4 M Li ^+^ as the only metal ion ^32^ and the hairpin ribozyme is fully active in Co(III) hexammine ions ^41– 43^, the HDV ribozyme is essentially inactive in either condition ^44^. This indicates a direct role for a metal in the catalytic mechanism. Nevertheless, the rate of cleavage by the HDV ribozyme depends upon pH ^44^ in a manner consistent with general acid–base catalysis. The genomic and antigenomic forms of the ribozyme adopt a double pseudoknot fold ^45, 46^. In these structures, there are no guanine nucleobases closer than 9 Å from the scissile phosphate. However, there is a cytosine nucleobase oriented so that its N3 points toward the scissile phosphate at a distance of 3.7 Å. Doudna *et al*. ^45^ suggested that C75 could act as a general base to catalyze cleavage. Bevilacqua ^44^ showed that the rate of cleavage of the HDV ribozyme increased log-linearly with pH up to 6.5 and remained at a plateau at higher pH values. The data fitted a p *K*_a_ of 6, potentially corresponding to a cytosine in an electronegative environment. However, in the absence of divalent cations, the pH dependence inverted ^44^. Since the rate fell with increasing pH, this indicates that C75 acts as the general acid. This was proven by Das and Piccirilli ^47^, who obtained almost full restoration of activity of a C-to-U variant when coupled with 5′-phosphorothiolate substitution. The bridging sulfur substitution creates a facile leaving group that no longer requires acid catalysis, consistent with cytosine acting as the general acid in the cleavage reaction. Taken together, the data suggest a model for catalysis in which a metal hydroxide acts as the general base and cytosine N3 acts as the general acid ( Figure 2C). The positions of these functionalities in available crystal structures ^45, 46^ are not fully consistent with their mechanistic roles, suggesting that the structures may not fully reflect the active conformation.

The TS ribozyme was identified by bioinformatic analysis and called twister-sister ^19^, and two structures have been solved in different forms ^48, 49^. The rate of cleavage of the ribozyme increases with pH up to neutrality, remaining at a plateau at higher pH, and increased log-linearly with Mg ^2+^ ion concentration ^19^. However, the activity was extremely low in either 1 M Li ^+^ or 1 mM Co(III) hexammine ions ^48^. Withdrawal of divalent cations had an effect similar to that in HDV, suggesting direct participation of a metal ion in the reaction. Importantly, the rate of cleavage was log-linearly dependent on the p *K*_a_ of the divalent metal ion present (that is, the ease of deprotonation of an inner-sphere water molecule) ^48^. This showed that a hydrated metal ion is involved in proton transfer. A metal ion directly bound to the cytosine immediately 5′ to the scissile phosphate was observed in the crystal ^48^, placing an inner-sphere water molecule close to the O2′nucleophile. However, the geometry around the scissile phosphate was far from in-line and it is likely that this structure would require significant reorganization to move into the active conformation. If the hydrated metal ion acts as a general base in cleavage, that leaves the question of what acts as a general acid. Mutagenesis suggests that modification of a cytosine (C7) results in the largest reduction of cleavage activity. In our crystal structure, C7 is 12 Å from the scissile phosphate and is involved in an H-bonded network. But remodeling the active center to create the required in-line geometry might also reposition C7, allowing it to participate in catalysis. Computational modeling has indicated that the geometry around the active center can be readily altered and that C7 can move closer to the scissile phosphate, whereupon it could either act as a general acid or bind a metal ion that acts as a general acid ^50^. In a more recent crystal structure of the TS ribozyme ^49^, the conformation about the scissile phosphate is quite different (though still far from in-line), consistent with the TS active center being quite malleable. Interestingly, in that structure, C7 is rather closer to the O5′ atom. Although TS remains one of the most incompletely understood ribozymes, the key catalytic participants are most probably a bound hydrated metal ion and a cytosine nucleobase. Thus, TS is provisionally placed with the HDV ribozyme as a discrete sub-group of the nucleolytic ribozymes ( Table 1). For both ribozymes, it is likely that the crystal structures are not fully reflective of the active conformations.

## G + O2′ and G + M ^2+^: the hammerhead and pistol ribozymes

It might not be immediately obvious that the hammerhead and pistol ribozymes should be discussed together, but there are striking structural and mechanistic similarities. Even comparison of the secondary structures and location of conserved nucleotides suggests a strong similarity between the two ribozymes ^51^. A new class of hammerhead ribozymes in which stems I and II interact through a pseudoknot has been described ^19^. A pseudoknot is also a key feature in the pistol ribozyme structure ^19, 52^, and if the two ribozyme secondary structures are rotated into the same frame, the strong similarity is apparent, and the positions of the conserved nucleotides and the scissile phosphate are very similar ^51^. Comparison of the three-dimensional structures in crystals of the hammerhead ^53^ and pistol ^51, 54, 55^ ribozymes also reveals a close similarity. If the pseudoknot interactions are stripped away, what remains is a three-way helical junction in both cases and the scissile phosphate is at the center. In both cases, the nucleotide 3′ to the scissile phosphate is base-paired to splay apart the 5′ and 3′ nucleotides, thereby generating the local geometry required for in-line attack. Furthermore, in both cases, there is a guanine nucleobase poised to remove the proton from the O2′ nucleophile. These (G12 in the hammerhead ^56, 57^ and G40 in the pistol ^51^) have been demonstrated to act as the general bases in the cleavage reactions.

This leaves the question of what serves as a general acid in these ribozymes. In neither structure is there a nucleobase close to the O5′ leaving group. However, in the hammerhead ribozyme, the O2′ of G8 is 3.2 Å from the O5′ atom and therefore could serve as the general acid. The p *K*_a_ of a 2′-OH group is very high, but computations of York *et al*. ^58^ suggested that a metal ion could move close and thus lower the p *K*_a_. Thomas and Perrin ^57^ showed that the reduced cleavage activity of a G8 O2′H variant could be restored by 5′-phosphorothiolate substitution at the scissile phosphate. This demonstrated the importance of the G8 O2′, but the effect could be indirect. The direct role of the G8 O2′ was demonstrated by substitution by 2′-amino ribose, whereupon the pH profile changed completely to a flattened bell shape ^51^. The modified ribozyme, rather like the hairpin or VS ribozymes, operates with one participant of high p *K*_a_ (G12 N1) and one of low p *K*_a_ (G8, 2′NH _2_).

**Figure 4.**
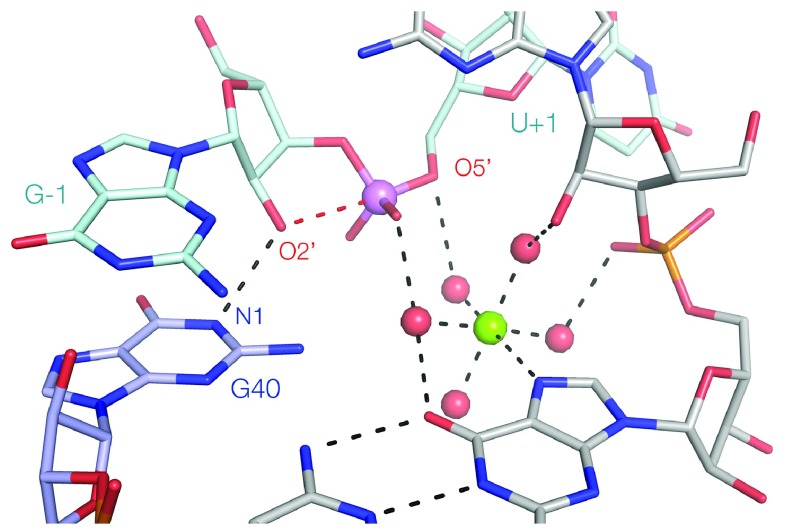
The proposed structure of the active site of the pistol ribozyme. This is derived from the crystal structure of the ribozyme ^51^. G40 acts as a general base, accepting a proton at N1 from the O2′ nucleophile. The green sphere is a Mg ^2+^ ion that is directly bound to N7 of the guanine nucleobase. The red spheres are inner-sphere water molecules, the positions of which have been modeled to maximize interactions with the RNA. The water molecule apical with N7 is well positioned to act as a general acid, protonating the O5′ leaving group.

When the same 2′NH _2_ substitution was performed at A32 in the pistol ribozyme (this lies in an analogous position to G8 in the hammerhead), a very different response was found. While absolute rates of cleavage were reduced, the shape of the pH profile was essentially unchanged from the unmodified ribozyme ^51^. Thus, unlike the hammerhead ribozyme, the corresponding 2′OH clearly does not act directly as the general acid. Instead, the available evidence indicates that pistol is the first ribozyme known to employ a hydrated metal ion as the general acid. The rate of cleavage increases in a log-linear manner with the p *K*_a_ of the metal ion used, consistent with an increase in reactivity of inner-sphere water molecules acting as a general acid ^51^. A metal ion is observed in the crystal structures of the pistol ribozyme ^51, 54^, directly bound to N7 of G33. Inner-sphere water molecules can be modeled so as to be bound to a number of active center functionalities, including the G32 O2′. The reaction rate is lowered if this O2′ is modified. The water molecule that is in the axial position with respect to G33 N7 is just 3.1 Å from the O5′ and thus is well positioned to act as the general acid ( Figure 4).

In many ways, the hammerhead and pistol ribozymes are very similar but ultimately they are mechanistically distinct. In terms of the general acid, they are in sense mirror images. The hammerhead uses an O2′ that is probably activated by a metal ion ( Figure 2D), whereas the pistol ribozyme uses a hydrated metal ion that is held in part by an O2′ ( Figure 2E).

## Some final perspectives

Two of the nucleolytic ribozymes have not been discussed. The structure of the *glm*S ribozyme ^17^ is known ^59, 60^ and it is another ribozyme that uses guanine N1 as the general base in cleavage ^61^. However, uniquely, the general acid is the amine of bound glucosamine-6-phosphate ( Figure 2F); that is, this ribozyme uses a coenzyme. At this time, the structure of the hatchet ribozyme is not known ^19^. However, biochemical data ^62^ are consistent with another metalloenzyme ribozyme of the HDV type.

Overall, we see that the use of nucleobases in proton transfers occurs frequently, and guanine is used as a general base in 6/8 ribozymes. However, the general acid is much more variable, and every possibility has been exploited in the group overall. Despite the limited catalytic resources of RNA, evolution has refined the use of various combinations of functional groups, hydrated metal ions, and even a coenzyme. But the expanding number of these ribozymes now allows these to be rationalized into a number of distinct mechanistic classes.
